# Exploring the Impact of Home-Based Serious Smartphone Resuscitation Gaming on Stress Among Nursing Students Practicing Simulated Adult Basic Life Support: Randomized Waitlist Controlled Trial

**DOI:** 10.2196/67623

**Published:** 2025-08-29

**Authors:** Nino Fijačko, Benjamin S Abella, Špela Metličar, Leon Kopitar, Robert Greif, Gregor Štiglic, Pavel Skok, Matej Strnad

**Affiliations:** 1 Faculty of Health Sciences, University of Maribor, Žitna 15, Maribor, 2000, Slovenia, 386 023004764; 2 University Medical Centre Maribor, Maribor, Slovenia; 3 Icahn School of Medicine at Mount Sinai, New York, NY, United States; 4 Medical Dispatch Centre Maribor, University Clinical Centre Ljubljana, Ljubljana, Slovenia; 5 Faculty of Electrical Engineering and Computer Science, University of Maribor, Maribor, Slovenia; 6 Faculty of Medicine, University of Bern, Bern, Switzerland; 7 Usher Institute, University of Edinburgh, Edinburgh, United Kingdom; 8 Faculty of Medicine, University of Maribor, Maribor, Slovenia; 9 Community Healthcare Centre Dr. Adolfa Drolca Maribor, Maribor, Slovenia

**Keywords:** adult basic life support, stress, serious games, gaming, nursing student, simulation, randomized controlled trial, RCT, smartphone

## Abstract

**Background:**

Simulation-based training is widely used in resuscitation education, yet limited research exists on how serious smartphone games—especially when used independently at home—impact stress levels during simulated adult basic life support (BLS). Understanding this relationship may offer new approaches to preparing health care students for high-stress clinical situations.

**Objective:**

This study aimed to evaluate the impact of a home-based serious resuscitation game, MOBICPR, on physiological stress markers among nursing students performing simulated adult BLS.

**Methods:**

In this single-center, randomized, waitlist controlled trial, 43 first-year nursing students were assigned to either an intervention group (IG) or a waitlist control group (WL-CG). Stress was measured at baseline and 2-week and 4-week follow-ups using electrodermal activity (EDA), blood volume pulse (BVP), heart rate (HR), and body temperature (BT) collected via the Empatica E4 wearable (Empatica Inc., USA). Each data collection point included 3 phases: mandala coloring before and after simulated adult BLS performance, and the adult BLS scenario itself. The MOBICPR game—a serious mobile game designed per the 2021 European Resuscitation Council adult BLS guidelines—was played at home over 2 weeks by IG (weeks 0‐2) and WL-CG (weeks 2‐4). A random forest classifier, trained on the AffectiveRoad dataset, predicted stress levels (none, moderate, and high) based on physiological signals.

**Results:**

Of 124 students invited, 43 participated (22 in IG, 21 in WL-CG; 38/43, 88% female; mean age of 19, SD 0.6 years). EDA, BVP, and BT significantly changed across measurement phases in both groups (*P*<.05), while HR did not show consistent differences (*P*>.05). Stress classification showed a significant decrease in stress after simulated adult BLS in the IG at the 2-week follow-up (*P*=.04), but not in the WL-CG. After 2 weeks of gameplay, 12 of 22 participants in the IG had lower stress levels after performing simulated adult BLS compared to before, suggesting an adaptive stress response. No significant group-level stress reductions were observed over time.

**Conclusions:**

Short-term, home-based gameplay using a serious resuscitation game modestly influenced physiological indicators of stress during simulated adult BLS among nursing students. While overall group stress levels remained stable, individualized responses suggested improved coping for some. Incorporating serious games into curricula could offer learners safe, gamified environments to rehearse stressful clinical scenarios. Future research should explore optimal game frequency and content depth to maximize educational and emotional resilience outcomes.

## Introduction

Adult basic life support (BLS) poses a substantial challenge for health care workers, as patient survival depends on their prompt and precise intervention [1]. To prepare future health care professionals for such challenges, educators can use various types of simulations [2], extended realities [3], or serious games [4]. According to Bergeron’s definition [5], serious games are “interactive computer applications, with or without a significant hardware component, like smartphones, that includes a challenging goal, is fun to play with, incorporates some concept of scoring, and imparts in the user a skill, knowledge, or attitude which can be applied in the real world.” Games are called serious when they have an educational purpose [6]. Playing a serious game on resuscitation of a patient in cardiac arrest can induce stress in users [4]. That might be positive, known as eustress, which arises as an individual’s response to situations within their manageable limits [7]. Conversely, negative stress, referred to as distress, emerges as an individual’s response to situations that surpass their ability to cope [8]. Serious smartphone games, as a component of mobile learning with gamification, have shown a positive impact on enhancing a range of learning outcomes and competencies in nursing as they offer the user the opportunity for repetition, which affects reduced stress levels and self-efficacy, and that within the home environment [9-13]. Beyond adult BLS [14-17], serious smartphone games have also been used to teach various clinical interventions, including advanced life support [18], choking management [19], triage [20], tactical combat casualty care [21], and a range of surgical [22-24] and nursing skills [25,26]. The assessment of stress during real or simulated resuscitation can be measured subjectively via a variety of scales [27]. Objectively through a range of electrophysiological metrics [28,29], including changes in heart rate [30,31], blood pressure [32], or skin temperature [33] alongside biomarkers (eg, cortisol [27,30] or α-amylase in saliva [32]). Over the past decade, new technology has improved our ability to track real-time stress levels using mobile phones and wearable devices [34]. While there are several studies on stress detection in controlled laboratory conditions [35-38], to our knowledge, no studies have examined perceived stress detection following the use of serious smartphone games based on cardiac arrest resuscitation content at home. That triggered us to investigate the effects of home-based serious smartphone gaming on stress among nursing students practicing simulated adult BLS.

## Methods

### Study Design

This single-center, simulated, randomized, waitlist controlled study was conducted at the Faculty of Health Sciences, University of Maribor (FHS UM) in Slovenia, Europe, from March to May 2023. A randomized, waitlisted, controlled study is a type of scientific experiment where participants are randomly assigned to either receive the intervention being tested immediately or be placed on a waiting list (control group) to receive the intervention after a delay, allowing for comparison of outcomes between the groups.

### Participants

Every nursing student registered in the first-degree program at the FHS UM, for the academic year 2022‐23 was invited to participate in the research project. A verbal presentation explained the research protocol to the students. They could sign up and provide their written consent. The inclusion criteria included nursing students who provided written informed consent, were 18 years or older, were capable of performing simulated adult BLS on a manikin (eg, without injury), and were willing to perform rescue breathing on a manikin. Notably, no exclusion criteria were set.

### Study Protocol

Using a computer-generated randomization list (Microsoft 365 Excel Enterprise), study participants were allocated in equal proportions to either the intervention group (IG) or the waitlist control group (WL-CG). The WL-CG participants were placed on a waitlist, receiving the intervention (playing the MOBICPR game at home for 2 weeks) after the IG. The evaluation of stress initially occurred at baseline and was followed up by 2 subsequent measurements: one after 2 weeks and another at week 4. Each of the 3 measurements (baseline and the 2 follow-ups) consisted of four phases: (phase 1) five minutes of coloring mandala at the study site; (phase 2) performing simulated adult BLS with 2 minutes of cardiopulmonary resuscitation (CPR) at the study site; (phase 3) five minutes of coloring mandala at the study site; and (phase 4) playing the MOBICPR game at the study site followed by 2 weeks at home, first for IG, and after 2 weeks for WL-CG. In the first 3 phases, stress was measured by using the Empatica E4 (Empatica Inc., USA) device.

The Empatica E4 device was used to measure stress, as it is a proven and reliable tool for assessing physiological stress indicators [39]. Its efficacy was established by comparing various physiological measures with those recorded by other devices under similar stressors. Notably, the Empatica E4 device demonstrated the highest accuracy in detecting stress compared to its counterparts [40].

**Figure 1.**
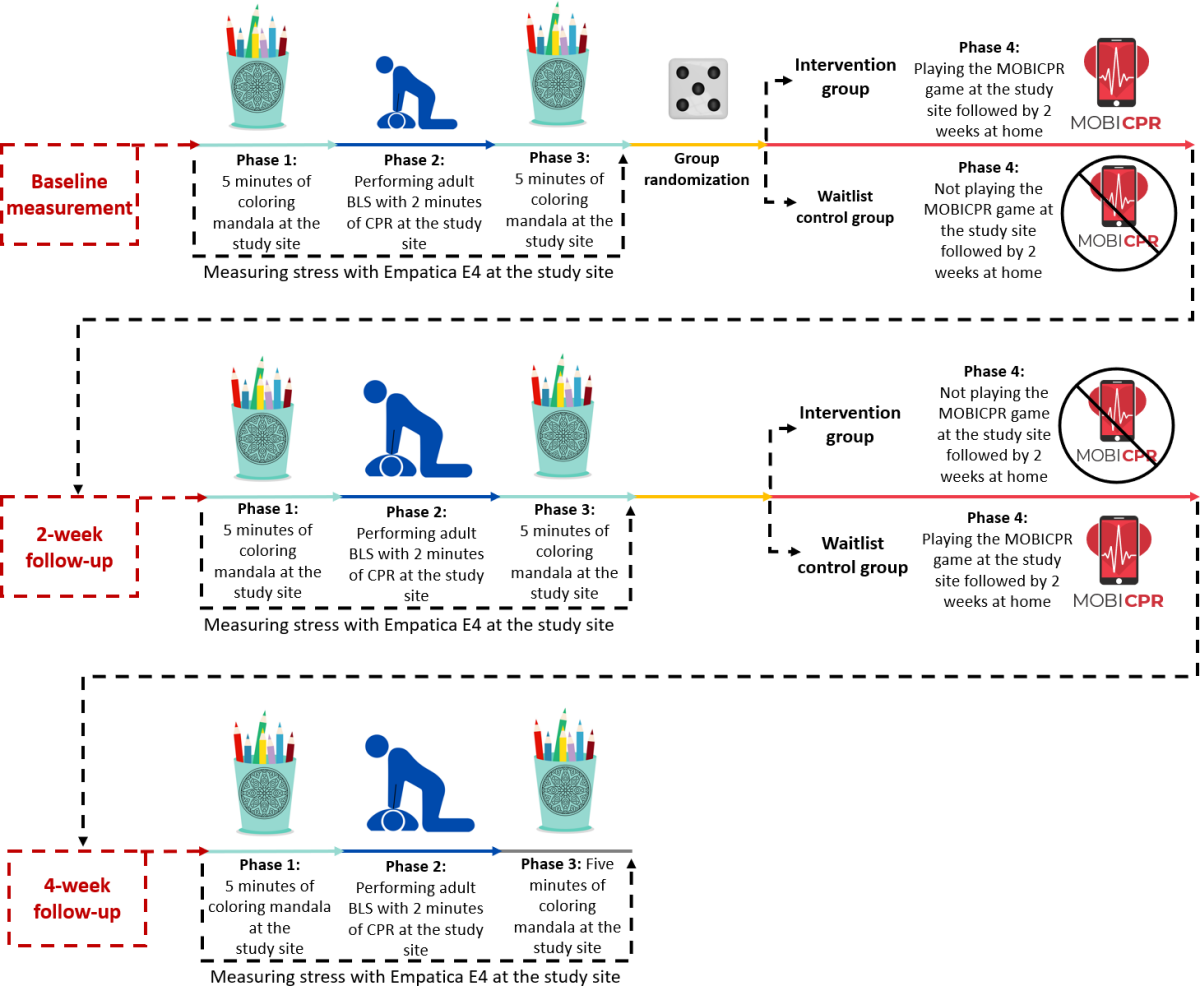
Study flowchart illustrating the randomized waitlist controlled trial design conducted at the Faculty of Health Sciences, University of Maribor, Slovenia (March-May 2023), evaluating the impact of home-based MOBICPR gameplay on physiological stress responses among nursing students performing simulated adult BLS. BLS: basic life support; CPR: cardiopulmonary resuscitation.

Before commencing the first phase of the measurement, the study investigator took a moment to reemphasize the study’s objectives to each nursing student and provided additional information to ensure clarity and understanding. In the first phase, each nursing student in both research groups selected a distinct mandala figure from the *Celestial Mandalas Coloring Book* [41] and marked it with their study number for identification. The inclusion of mandala coloring was primarily designed to divert nursing students’ attention away from the realization that they were undergoing stress measurement. This approach is grounded in the concept that structured coloring activities, particularly those involving predesigned repetitive patterns such as mandalas, are associated with mindfulness practices and contribute to calming the mind and reducing perceived stress levels [42]. Before coloring the mandala figure for 5 minutes, the investigator securely placed the Empatica E4 device on the nondominant hand of the study participant by following Empatica instructions and connected the device to the E4 real-time application (Empatica Inc., USA) installed on a Google Pixel 4 (Google LLC, USA) smartphone to facilitate real-time monitoring and data recording. The coloring activity took place in a separate quiet room without interruptions, where a steady temperature of 21 to 23 °C was maintained. In the second phase, each study participant was given a scenario of out-of-hospital cardiac arrest (OHCA) to read. Then they proceeded to a designated room where they performed simulated adult BLS with 2 minutes of CPR on an adult manikin (Resusci Anne QCPR, Laerdal Medical, Norway) using an automated external defibrillator (Trainer AED, Defibtech LLC, USA). The OHCA scenario was performed in a staged kitchen, which was surrounded by mobile walls in the hospital’s simulated room [43,44]. In the third phase, the study participants continued coloring preselected mandalas. After 5 minutes, the investigator removed the Empatica E4 device. After the third phase, nursing students played the MOBICPR game under the investigator’s observation to familiarize themselves with the game’s mechanics and content correctly. The game’s content (eg, the 5-finger mnemonic), which guided the user through OHCA, was developed through the Delphi process [45] and adhered to the 2021 European Resuscitation Council adult BLS guidelines [46]. The MOBICPR game decreases the victim’s survival probability with each error made by the player. Upon completion, the MOBICPR game provided a cumulative score as a gamification element, indicating the victim’s likelihood of survival. A score above 51% suggests a successful outcome [47]. Playing the MOBICPR game at home has been shown to enhance and maintain theoretical knowledge of adult BLS, although it did not improve practical skills [43]. The average gameplay duration, based on previous studies, was 8 minutes. Subsequently, the IG received a Samsung Galaxy A13 (Samsung Group, South Korea) smartphone, preloaded with the MOBICPR game. Participants were instructed to play the game at home for 2 weeks, as frequently as they desired. After those 2 weeks, nursing students in the IG returned the smartphones. Subsequently, these smartphones were handed over to the study participants in WL-CG to play the MOBICPR game at home for 2 weeks. Then they returned the smartphones too (Figure 1).

### Outcome

The primary outcome of the study was to assess whether playing the MOBICPR game at home for 2 weeks led to changes in physiological metrics among nursing students in the IG and WL-CG before and after performing simulated adult BLS on a manikin, with stress levels evaluated as a secondary outcome.

### Collecting Data, Measuring Stress, and Statistical Analysis

**Figure 2.**
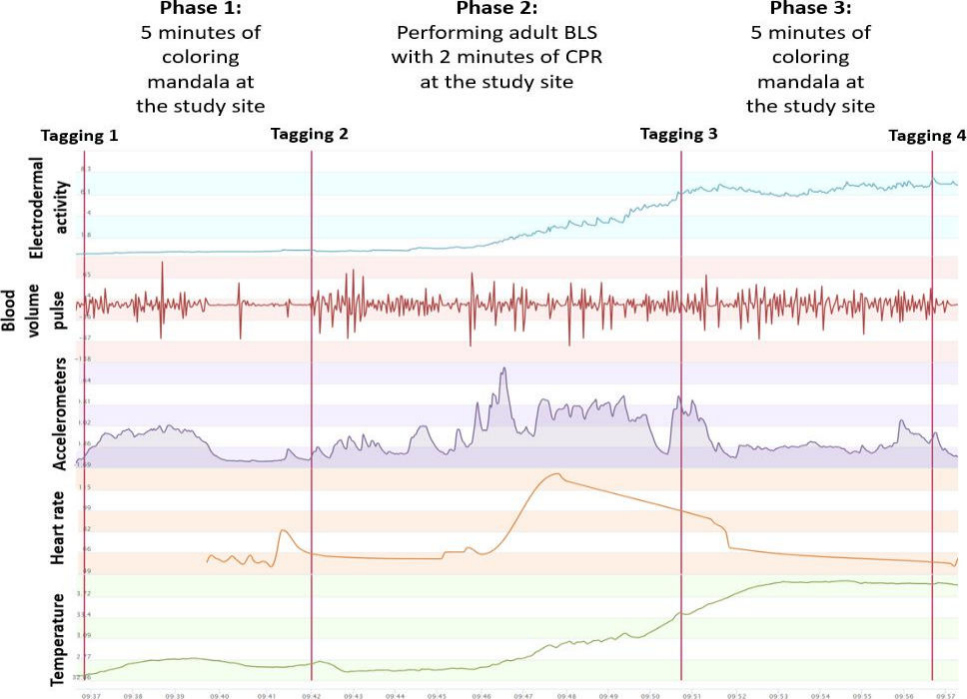
Example of Empatica E4 “tagging” used for stress measurement, showing data collection phases for one nursing student in the randomized waitlist controlled trial on the effects of serious gaming on stress during simulated adult BLS in Slovenia (2023). Phases 1 and 3 represent 5-minute mandala coloring periods before and after simulated adult BLS. BLS: basic life support; CPR: cardiopulmonary resuscitation.

To signify the beginning and the end of each phase for every study participant, the investigator was required to activate a feature on the Empatica E4 device by pressing a button, a process known as “tagging.” Each study participant was assigned 4 distinct tags: the first tag signified the start of phase 1, the second marked the conclusion of phase 1 and the commencement of the second phase, the third tag indicated the end of the second phase and the beginning of the third, and the fourth tag represented the conclusion of the third phase. Once the fourth tag was logged, the Empatica E4 device was removed from the nursing student’s wrist. Before the Empatica E4 device was turned off, the data were transferred to the Empatica Manager software via the Bluetooth built into the Empatica E4 device. For each nursing student in the study, 3 separate recordings were taken using the Empatica E4 device—at baseline and then after 2 and 4 weeks. These recordings measured five distinct physiological metrics: (1) electrodermal activity (EDA), (2) blood volume pulse (BVP), (3) accelerometer data, (4) heart rate (HR), and (5) body temperature (BT), as shown in Figure 2.

Statistical analyses were carried out over a period extending from May 2023 to September 2024. The research was conducted in the programming language R (R Foundation for Statistical Computing, Austria). We read Empatica E4 device physiological metrics with a library “wearables” [48], while a stacked bar graph was generated using a library ggplot2 [49].

The age of participants was the only measure where we used mean and SD. For physiological metrics, we first assessed normality using the Shapiro-Wilk test. Data with *P* values ≥.05 were considered normally distributed and analyzed with parametric tests (eg, Student *t* test), while nonnormal data (*P*<.05) were analyzed using nonparametric alternatives (eg, Mann-Whitney *U* test for independent samples or Wilcoxon signed-rank test for paired comparisons).

### Primary Outcome

We focused on the measurement of 4 key physiological metrics: EDA, BT, BVP, and HR. Accelerometer data were excluded, as participants wore the Empatica E4 on their nondominant hand, which remained at rest during the mandala coloring activity, consistent with procedures followed in similar studies [35,39]. We analyzed those physiological metrics by comparing the average values along with their specified 95% CIs and relevant statistical tests. To address multiple comparisons in the pairwise analysis of psychological measures, we applied a false discovery rate adjustment to minimize the risk of false positives.

### Secondary Outcome

We used physiological metrics (EDA, BT, and HR) to measure stress levels, implementing the pretrained random forest classifier, one of the most widely used machine learning techniques [50], and was developed by Hosseini et al [33]. The classifier was trained on the AffectiveRoad dataset [51], optimized through grid search and cross-validation, and categorized stress into three levels: 0 (no stress), 1 (moderate stress), and 2 (high stress). This classification was based on thresholds from the AffectiveRoad dataset, where *S*≤0.65 indicated no stress, 0.65≤*S*≤1.3 indicated moderate stress, and *S*≥1.3 indicated high stress.

The classifier extracted 48 signal features from a 10-second sliding window of EDA, BT, and HR data, along with contextual features from the preceding 10 windows. While we did not contribute to the classifier’s training or the categorization process, we calculated the median stress value for each measurement, leading to occasional intermediate values like 0.5 (between no stress and moderate stress) and 1.5 (between moderate and high stress).

Stress level prediction was determined using the median value. The median was selected because, in a 5-minute session, predicted stress levels could display an imbalanced distribution (eg, [2, 2, 2, 1, 0]). Using the median was preferred over the mean when predicted values did not adhere to a normal distribution [52]. The Wilcoxon signed-rank test was used to assess the statistical significance of pairwise differences in stress levels at different time points. To address multiple comparisons in the pairwise analysis of psychological measures, we applied a false discovery rate adjustment to minimize the risk of false positives.

We also conducted pairwise comparisons of the MOBICPR game’s effect on stress levels during home use, examining positive, negative, and equal ranks to represent how stress levels changed in phase 1 (before performing simulated adult BLS) and phase 3 (after performing simulated adult BLS). A positive rank indicated increased stress (ie, the number of times stress levels increased), negative ranks indicated decreased stress (ie, the number of times stress levels decreased), and equal ranks indicated no change in stress. The Wilcoxon signed-rank test was used to inspect the significant differences in the ranks of stress levels before and after simulated adult BLS.

All tests were 2-tailed, and statistical significance was set at *P*<.05, with *P* values adjusted where appropriate using the Benjamini-Hochberg false discovery rate procedure.

### Ethical Considerations

This study was reviewed and approved by the Slovenian National Medical Ethics Committee (approval number 0120-157/2018) and registered at ClinicalTrials.gov (NCT05784675). Due to the complexity of the registered study protocol, the publication was planned in multiple parts; this study represents one of two main research papers derived from the original design. The study protocol was written in accordance with the CONSORT-EHEALTH (Consolidated Standards of Reporting Trials of Electronic and Mobile Health Applications and Online Telehealth) checklist [53]. All participants received both verbal and written information about the study and provided written informed consent prior to enrollment. Strict measures were implemented to ensure confidentiality and data protection; all data were anonymized prior to analysis, and no identifying personal information was stored or included in publication materials. Participation was entirely voluntary and not linked to academic performance or evaluation. No images or supplementary materials contain identifiable information about participants, and thus no additional image consent is required. Participants were rewarded for their participation with a free beverage from a vending machine and a printed copy of the Game Changer painting by the street artist Banksy.

## Results

### Participant Details

**Figure 3.**
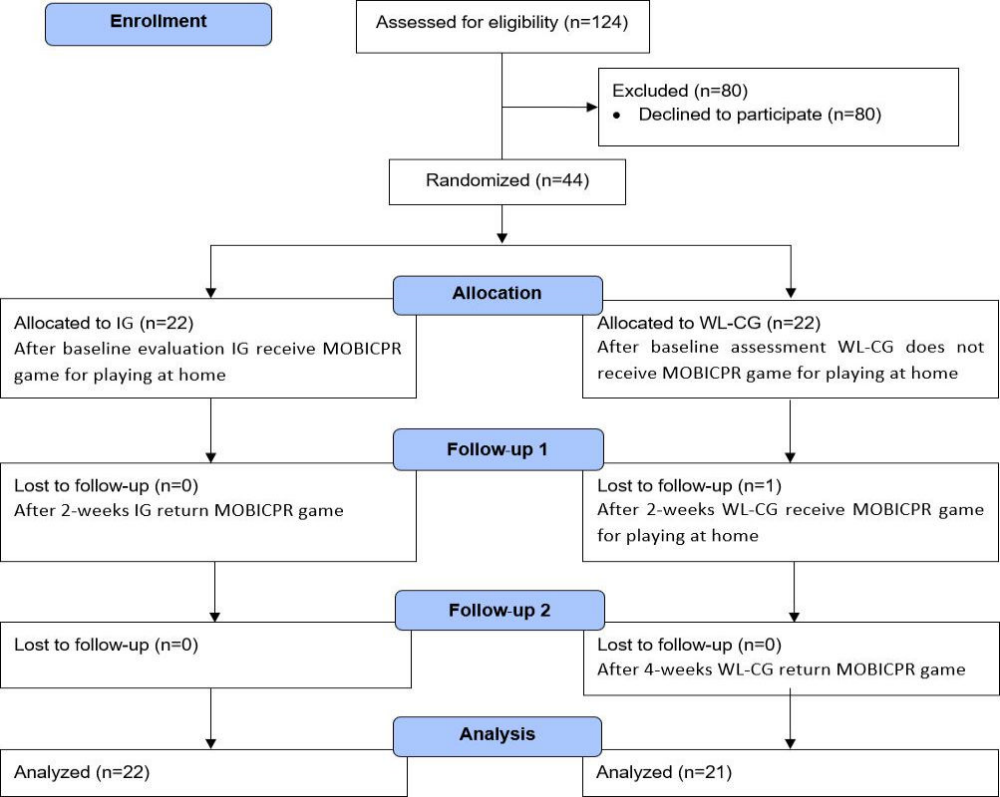
Flow diagram of participant enrollment and group assignment in a randomized waitlist controlled trial with Slovenian nursing students (n=43), evaluating physiological stress responses to serious game-based adult BLS training using Empatica E4 wearables (March-May 2023). BLS: basic life support; IG: intervention group; WL-CG: waitlist control group.

Out of 124 nursing students at the enrollment, 44 (35.5%) participated in the study. At follow-up, 1 nursing student in the WL-CG dropped out. In the end, 43 study participants were included in the final analysis, 22 in IG and 21 in WL-CG (Figure 3).

Most were women (38/43, 88%) with a mean age of 19 (SD 0.6) years. On average, study participants in both groups played the MOBICPR game at home 7 (SD 4) times.

### Primary Outcome

First, the average values for the physiological metrics EDA (μS), BVP, HR (beats/minute), and BT (°C) were calculated. BVP represents relative changes in blood flow and is typically expressed without a specific unit [54]. EDA, BVP, and BT show statistically significant changes across measurement phases in both IG and WL-CG, with *P* values below .05 in multiple comparisons. In contrast, HR shows no consistent statistical significance, as most *P* values are above .05, indicating minimal differences between IG and WL-CG in multiple comparisons (Table 1). Overall, there were few statistically significant differences between IG and WL-CG across measurement phases (*P*>.05).

**Table 1.** Comparison of physiological metrics (electrodermal activity, blood volume pulse, heart rate, and body temperature) across measurement phases in Slovenian nursing students during a randomized waitlist controlled trial on stress and serious smartphone gaming in adult basic life support training, conducted March-May 2023. All metrics were measured using the Empatica E4 device.

Physiological metrics and measurement (phase)	Intervention group, mean (95% CI)	Waitlist control group, mean (95% CI)	*P* value^a^
Electrodermal activity (μS)			
Baseline			
Baseline (phase 1)	1.69 (0.56‐2.82)	1.42 (0.47‐2.36)	.98
Baseline (phase 3)	6.5 (3.3‐9.69)	7.41 (4.85‐9.97)	.51
*P* value^b^	.002	<.001	
2-week follow-up			
2-week follow-up (phase 1)	1.96 (0.45‐3.46)	1.71 (0.5‐2.91)	.37
2-week follow-up (phase 3)	6.85 (3.82‐9.88)	8.54 (5.6‐11.48)	.34
*P* value^b^	<.001	<.001	
4-week follow-up			
4-week follow-up (phase 1)	1.89 (-0.06‐3.85)	0.64 (-0.02‐1.3)	.84
4-week follow-up (phase 3)	8.01 (3.77‐12.24)	7.7 (5.28‐10.13)	.49
*P* value^b^	<.001	<.001	
Blood volume pulse			
Baseline			
Baseline (phase 1)	30.44 (22.02‐38.87)	23.07 (16.74‐29.39)	.29
Baseline (phase 3)	50.62 (37.74‐63.49)	48.58 (37.21‐59.94)	>.99
*P* value^b^	.02	<.001	
2-week follow-up			
2-week follow-up (phase 1)	22.39 (17.05‐27.73)	23.55 (17.7‐29.41)	.62
2-week follow-up (phase 3)	44.98 (32.02‐57.93)	41.27 (32.26‐50.28)	.97
*P* value^b^	<.001	<.001	
4-week follow-up			
4-week follow-up (phase 1)	26.7 (20.01‐33.4)	26.07 (20.62‐31.53)	.99
4-week follow-up (phase 3)	41.6 (32.76‐50.43)	45.52 (36.05‐54.98)	.53
*P* value^b^	.007	<.001	
Heart rate (beats/minute)			
Baseline			
Baseline (phase 1)	87.53 (81.89‐93.16)	86.88 (82.03‐91.73)	.86
Baseline (phase 3)	86.85 (79.41‐94.29)	88.78 (82.91‐94.66)	.67
*P* value^b^	.77	.36	
2-week follow-up			
2-week follow-up (phase 1)	84.05 (78.7‐89.41)	86.35 (81.59‐91.11)	.76
2-week follow-up (phase 3)	85.54 (80.49‐90.59)	92.55 (87.51‐97.58)	.06
*P* value^b^	.45	.002	
4-week follow-up			
4-week follow-up (phase 1)	84.59 (79.5‐89.68)	87.21 (80.72‐93.69)	.51
4-week follow-up (phase 3)	86.8 (81.7‐91.91)	92.88 (86.68‐99.09)	.12
*P* value^b^	.19	.06	
Body temperature (°C)			
Baseline			
Baseline (phase 1)	32.7 (32.27‐33.13)	31.73 (30.95‐32.51)	.03
Baseline (phase 3)	33.32 (32.75‐33.89)	32.73 (31.96‐33.5)	.20
*P* value^b^	.005	<.001	
2-week follow-up			
2-week follow-up (phase 1)	32.22 (31.54‐32.89)	32.71 (32.02‐33.4)	.30
2-week follow-up (phase 3)	32.94 (32.12‐33.76)	33.14 (32.34‐33.94)	>.99
*P* value^b^	.003	.05	
4-week follow-up			
4-week follow-up (phase 1)	32.52 (31.82‐33.23)	32.45 (31.38‐33.52)	.55
4-week follow-up (phase 3)	33.25 (32.43‐34.06)	33.02 (32.11‐33.92)	.48
*P* value^b^	.02	.03	

a These *P* values indicate between-group comparisons (intervention group vs waitlist control group) at the same timepoint (baseline or 2-week follow-up or 4-week follow-up).

b These *P* values represent within-group comparisons across phases (e.g. Phase 1 vs. Phase 3).

### Secondary Outcome

Additionally, stress levels were assessed using the physiological metrics EDA, BT, and HR. In phase 1, the IG showed a significant within-group change between baseline and the 2-week follow-up (*P*=.04). In contrast, no significant changes were observed in the WL-CG. In contrast, no significant changes were found in the WL-CG. Other comparisons between the IG and WL-CG, as well as across follow-up points, did not show significant differences (Table 2).

**Table 2.** Average stress level comparisons between pre- and postadult basic life support mandala coloring phases in intervention and control groups across 3 time points (baseline, 2-week, and 4-week follow-ups) in a 2023 study of MOBICPR game use among Slovenian nursing students.

Measurement (phase)	Intervention group (n=22), mean	Waitlist control group (n=21), mean	*P* value^a^
Baseline			
Baseline (phase 1)	0.75	0.74	.96
Baseline (phase 3)	0.71	1.09	.18
*P* value^b^	.22	.22	
2-week follow-up			
2-week follow-up (phase 1)	0.61	0.57	.69
2-week follow-up (phase 3)	1.11	0.76	.12
*P* value^b^	.04	.45	
4-week follow-up			
4-week follow-up (phase 1)	0.66	0.45	.26
4-week follow-up (phase 3)	0.68	0.81	.56
*P* value^b^	.81	.16	

a These *P* values represent between-group comparisons at each timepoint.

b These *P* values represent within-group phase comparisons.

Table 3 compares the ranks before and after simulated adult BLS training between the IG (n=22) and WL-CG (n=21) at 2 different points: baseline and after 2 weeks of playing the MOBICPR game at home. At baseline, 8 participants in the IG had a negative rank, 7 had a positive rank, and 7 had an equal rank, with a *P* value of 0.77, indicating no significant difference. In the WL-CG, 6 participants had a negative rank, 9 had a positive rank, and 6 had an equal rank, with a *P* value of 0.45, also showing no significant difference. After 2 weeks of playing the MOBICPR game at home, the IG showed a shift, with 6 participants having a negative rank, 12 having a positive rank, and 4 having an equal rank, with a *P* value of .04, suggesting a significant change. In the WL-CG, 4 participants had a negative rank, 10 had a positive rank, and 7 had an equal rank, with a *P* value of .16, indicating no significant difference.

**Table 3.** Pairwise comparison of stress level changes before and after adult basic life support performance, measured before and after 2 weeks of home MOBICPR gameplay in a 2023 randomized trial of Slovenian nursing students using wearable stress data.

	Intervention group (n=22)		Waitlist control group (n=21)	
Measurement (phase) and rank before and after adult basic life support	Values, n	*P* value	Values, n	*P* value
Baseline		.77		.45
Negative rank	8		6	
Positive rank	7		9	
Equal rank	7		6	
After 2 weeks of playing MOBICPR at home^a^		.04		.16
Negative rank	6		4	
Positive rank	12		10	
Equal rank	4		7	

a “After 2 weeks of playing MOBICPR at home” refers to the measurement taken 2 weeks after baseline for the intervention group and 4 weeks after baseline for the waitlist control group, reflecting the point at which each group completed 2 weeks of home gameplay.

**Figure 4.**
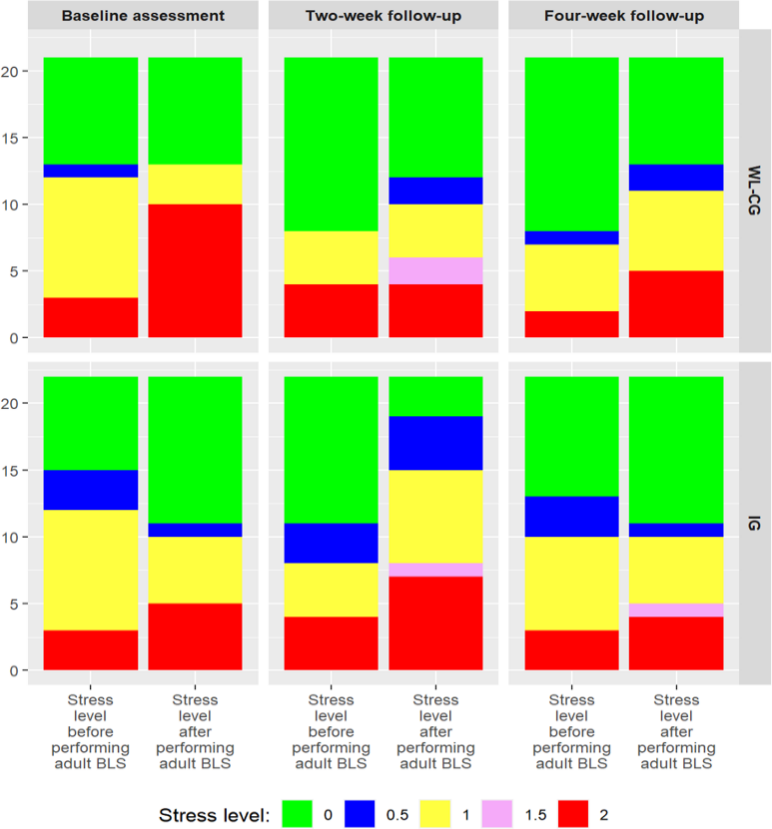
Stress level distribution before and after simulated adult BLS among nursing students in the intervention and waitlist control groups at baseline, 2-week, and 4-week follow-ups in a 2023 randomized trial using the MOBICPR game and Empatica E4 wearable sensors. BLS: basic life support; IG: intervention group; WL-CG: waitlist control group.

At baseline, both groups show a similar distribution of stress levels, with many participants in both groups having stress levels of 0 and 1 before performing simulated adult BLS. There is a noticeable shift in stress levels after performing simulated adult BLS, with more participants reporting higher stress levels (especially stress level 2). During the 2-week follow-up, stress levels before performing simulated adult BLS show a larger distribution of lower stress levels (0 and 0.5) in both groups, but after performing simulated adult BLS, the IG shows a slight shift toward lower stress levels, while the WL-CG still shows a higher proportion of participants experiencing stress level 2. During the 4-week follow-up, stress levels before and after performing simulated adult BLS in the IG appear more balanced, with many participants maintaining lower stress levels (0 and 0.5). In the WL-CG, there is still a shift toward higher stress levels after performing adult BLS, although there is a slight reduction in stress level 2 compared to previous time points (Figure 4).

## Discussion

### Principal Findings

To our knowledge, this study is the first to examine the effects of home-based serious smartphone resuscitation gaming on stress levels, measured using the Empatica E4 device, among nursing students participating in simulated adult BLS. Research using methodologies like ours has mainly focused on assessing cognitive, behavioral, and motivational learning outcomes in educational settings [9-13], while dedicating less attention to the evaluation of stress levels. A 2017 study measured the impact of team leader stress on teams practicing advanced life support in a simulation environment, using the Empatica E4 device and self-reported measures for assessment [55]. A key finding was that EDA levels increased as team performance improved and decreased with poorer performance. This aligns partially with our findings, where EDA, BVP, and BT showed statistically significant changes between groups (IG and WL-CG) and across measurement phases, indicating that these physiological metrics are sensitive to changes over time and may reflect varying levels of stress or engagement. Stress is known to elicit physiological responses, including elevated HR, particularly in simulation environments [56]. Studies conducted in clinical environments with nurses in intensive care using the Empatica E4 device to measure stress have shown positive correlations between stress and HR, stress and BT, as well as between HR and EDA [33,57,58]. Similarly, elevated HR has also been observed in simulation environments [56]. Our findings regarding HR revealed no consistent statistically significant changes across measurement phases or between groups, which contrasts with prior studies conducted in both clinical and simulation environments. For example, Ahmadi et al [57] and Zhang et al [58] demonstrated positive correlations between HR and stress among intensive care unit nurses during actual patient care, and Harvey et al [56] found similar results in simulated trauma scenarios. These discrepancies may stem from differences in context, measurement duration, and task complexity. While those studies involved longer monitoring periods (up to 12 hours) and real or high-fidelity simulations with greater cognitive load, our study involved short-term simulations (~10 minutes) using manikins and a controlled environment. Furthermore, HR is known to be influenced by various confounding factors such as baseline fitness, breathing patterns, and individual stress perception, which may reduce its sensitivity in short-duration tasks like ours. Thus, while EDA, BVP, and BT showed consistent responses, HR alone may not be a reliable standalone indicator of acute stress in brief, low-to-moderate intensity simulation-based tasks. These contrasting results highlight the importance of multimodal stress measurement in educational research, especially when using wearables in short simulated scenarios. Regarding BT, measured on participants’ forearms, our study reported similar values to those observed in a study involving 3 different cognitive load tasks [59], with temperatures ranging between 31 and 33 °C. These results align with the normal regional variation of human skin temperature, which ranges from 29.8 (SD 1.6) °C on the sole to 34.7 (SD 0.6) °C on the neck and 32.6 (SD 0.9) °C on the forearm [60]. Both studies measured temperatures in similar environments; however, they used a digital thermometer, while we used the Empatica E4 device.

The term “stress paradox” refers to the phenomenon where exposure to a task induces stress, which, counterintuitively, can have a positive effect (eustress) on the learning curve by enhancing performance and fostering growth. In our study, this paradox is illustrated by the physiological response patterns observed. EDA, BVP, and BT showed changes across measurement phases, indicating engagement and potential arousal during simulated adult BLS performance. Interestingly, despite participants playing the MOBICPR game at home an average of less than 8 times, stress levels did not change significantly at the group level, as shown in both absolute values and machine learning–derived stress classifications. However, performance in the simulated adult BLS scenario improved over time, particularly in theoretical knowledge retention as reported in our earlier publication [43]. Moreover, we observed a statistically significant shift in stress response within the IG after 2 weeks of gameplay: 12 participants showed reduced stress after performing simulated adult BLS compared to before. This pattern supports the idea that controlled exposure to stress via serious gaming may lead to adaptive physiological responses without overwhelming distress, aligning with prior research that Slovenian nurses often manage stress well during resuscitation due to repeated simulation training [61,62]. Thus, even in the absence of reduced stress at a group level, individual adaptation and performance improvement hint at the beneficial effect of eustress within the framework of the stress paradox. That feeling of eustress benefits learning and leads to “stress-related growth” [63].

Gamification, incorporated into a serious game, allows users to learn and retain content through mobile learning, as it represents a familiar educational environment for the modern user [64]. However, the open market currently provides a limited selection of serious smartphone games specifically tailored for teaching adults [65]. Moreover, several serious smartphone games used in recent studies are no longer available [9-13,66]. By developing the MOBICPR game, we aim to address the current scarcity of accessible serious smartphone games for teaching adult BLS by offering a freely available tool for both mobile [67] and web-based learning [18,68]. The MOBICPR game will be freely available in 2026 following the release of the new European Resuscitation Council adult BLS guidelines. It will be published on the Google Play Store and can also be requested directly from the corresponding author as an Android (.apk) file. Dissemination will include collaboration with academic institutions, promotion at conferences, participation in innovation competitions and award submissions, as well as targeted outreach via social media platforms. Initially, the game will be in English and compatible with Android devices. Language localization and iOS support are planned for future updates, pending available resources. Additionally, our approach incorporates heutagogy, where learners have the autonomy to shape their own learning experience, a method that promises to redefine future educational practices. By integrating gamification features into the MOBICPR game, our goal is for users to engage with it more frequently, thereby enhancing the retention of knowledge over a longer period [43,47]. Additionally, a 2023 study suggests that playing console games for approximately 20 hours over 14 days can enhance endoscopic skills [69]. In contrast, our participants played an average of 7 times during the same period, totaling about 45 minutes. This reduced gameplay duration could potentially impact their level of stress. Although immersive educational methods such as virtual reality hold promise for impacting stress levels, recent research on both adult and pediatric basic and advanced life support presents mixed outcomes, indicating the need for further studies [30,70].

A recent review identified several stress mitigators that could influence stress and decision-making during resuscitation, including cognitive aids such as checklists, stress management training, and meditation [71]. Specifically, meditation involving the use of mandalas, like our approach, may support health care professionals in managing stress. Recent studies suggest that mandalas are linked to mindfulness practices, helping nurses working in critical care to calm the mind and reduce perceived stress levels [42]. Similarly, activities like Sudoku or daydreaming can also have a similar effect on reducing stress [59].

### Limitations

Our studies have several limitations. First, although we invited all nursing students enrolled in the 2022/2023 academic year to participate, only 44 chose to enroll, and 1 dropped out, resulting in a final sample size of 43 participants. While this sample size is comparable to other studies [33,57,58] using the Empatica E4 device for stress assessment, it remains relatively small, which may limit the statistical power and generalizability of our findings. No formal a priori power analysis was conducted due to the exploratory nature of this study and the absence of comparable effect sizes from similar interventions. Future research should include a formal power calculation and a larger, more diverse sample to validate and extend the results presented here. Looking ahead, we could engage a younger audience to determine if the MOBICPR game is effective in teaching them adult BLS content at home prior to participating in actual training sessions. Additionally, the sample size of participants could have been larger if we had the ability to offer incentives, as is done at other universities [72-74]. Despite this, our retention rate remained steady, with no participants dropping out as the study progressed. Second, we have only one Empatica E4 device, which influences the process of collecting data. It happened that event marking was inconsistent, causing event mark times to appear either too close to each other or separated from each other to a large extent. This was resolved by eliminating intermittent tags if the time duration between 2 event marks did not exceed 4.5 minutes. We allowed a 30-second variation since recording the event can hardly be done to a second precisely. This means the first event mark indicated the beginning of the first phase, followed by an event mark recorded at least 4.5 minutes later, indicating the time span of the first phase. Third, due to the limitations of the Empatica E4 device, we were unable to record data during phase 2, where simulated adult BLS was performed, because of hand movements. A more convenient way to accurately measure stress during physical activity may involve the use of electroencephalogram headbands [75] or wireless electrocardiogram monitors [76]. With advancements in artificial intelligence (ie, deep learning), stress detection based on facial expressions has also become increasingly feasible [77]. Future studies could explore these alternative technologies to enhance data collection during physically active scenarios. Fourth, having study participants undertake the same simulated adult BLS scenario may have made them more familiar with it, thereby potentially reducing their stress levels due to repetition and anticipation of what was coming next. Similarly, the limited content and structure of the MOBICPR game may have contributed to decreased cognitive load over time. Although this familiarity reflects the positive effects of repeated practice, it may also have muted the variability in stress responses we aimed to capture. Adding more diverse and randomized scenarios could help reduce this effect, but such improvements were not feasible within the scope of this study due to financial constraints. Fifth, many of the study participants were female, which could introduce gender bias and influence the results.

### Conclusions

Our study has shown that playing a serious smartphone game such as MOBICPR game at home can have an impact on stress levels before and after performing adult BLS. The application of serious gaming and its incorporation into the educational curriculum might also be considered more widely. That might give users the opportunity to practice in a safe environment like home, without risk of harm, and empower them for tomorrow’s challenges.

## Supplementary material

10.2196/67623 Checklist 1 CONSORT-EHEALTH checklist (v 1.6.1).

## Abbreviations

BLS: basic life support
BT: body temperature
BVP: blood volume pulse
CONSORT-EHEALTH: Consolidated Standards of Reporting Trials of Electronic and Mobile Health Applications and Online Telehealth
CPR: cardiopulmonary resuscitation
EDA: electrodermal activity
HR: heart rate
IG: intervention group
OHCA: out-of-hospital cardiac arrest
WL-CG: waitlist control group
